# Interrogating the lack of diversity of thought in the pandemic response that led to mistakes - holistic evidence-based approach to deal with future pandemics

**DOI:** 10.3389/fpubh.2023.1310210

**Published:** 2023-12-21

**Authors:** Colleen Aldous, Hendrik G. Kruger

**Affiliations:** ^1^Nelson R Mandela School of Clinical Medicine, University of KwaZulu-Natal, Durban, South Africa; ^2^School of Health Sciences, University of Kwazulu-Natal, Durban, South Africa

**Keywords:** pandemic management, lockdowns, evidence based medicine, COVID-19, holistic health, holistic management

## Abstract

The COVID-19 pandemic, triggered by the severe acute respiratory syndrome coronavirus 2 (SARS-CoV-2), rapidly became a worldwide emergency. How it was managed garnered both commendation and vehement censure. This crisis profoundly affected healthcare, the economy, education, and public confidence in scientific endeavors. Our primary aim was to scrutinize the shortcomings in the pandemic management and to articulate a more effective strategy for handling prospective pandemics. We delved into the errors encountered in the COVID-19 response and posited a holistic, evidence-grounded approach for future pandemic mitigation.

## Introduction

In March 2020, the World Health Organization declared COVID-19 a global pandemic (1, 2). As of July 2023, there were 768 million reported cases and approximately 6.95 million COVID-19 fatalities (3). Subsequent waves, driven by variants such as Omicron, have further complicated an already uncertain situation, (4) as there was a growing concern about the potential diminished efficacy of vaccines (5).

Evaluating the global endeavors to contain the virus is paramount, informing future strategies and commemorating effective actions learned from past experiences. While regulatory bodies and public health experts have celebrated various interventions as successful, thoroughly examining their limitations is essential for enhancing future pandemic preparedness. Both governmental agencies and healthcare organizations must meticulously review their responses to formulate more effective public health policies.

A critical analysis encompassing a range of interventions and their subsequent outcomes, spanning health-related and economic dimensions, promises invaluable insights. Such an assessment can serve as a roadmap for optimizing future crisis response strategies and reducing the likelihood of repeating past mistakes. This paper briefly surveys the lessons that should have been drawn from previous respiratory virus pandemics. We then delve into the numerous deficiencies observed in our response to the COVID-19 pandemic, offering recommendations for a forward-looking approach that emphasizes a shift in pandemic response leadership from a clinician-centric model to a multidisciplinary one.

### What we have learned from previous flu pandemics

Over the last century and beyond, pandemics such as the Spanish flu (1918), (6) SARS-Cov-1 in 2003 (7) and the Middle East respiratory syndrome (MERS) in 2012 (8) have occurred. However, SARS-Cov-1 and MERS were transmitted largely through close contacts (9–11) and these pandemics were relatively less severe. Spanish flu and COVID-19 are also spreaded via airborne transmission (12, 13).

Although SARS-Covid-1 is also transmitted via airborne particles, it appears not enough studies have been done to determine the exact mechanism for airborne transmission (14). COVID-19 (SARS-Co2-2) transmission in aeroplanes is similar to that of the influenza A(H1N1)virus, in that transmission occurs mainly via a short-range airborne route.

The reproductive number (R) for the Spanish Flu (15) was in a similar range to that of COVID-19 (16–19), although the 1918 pandemic was significantly more deadly, given that post-World War I, food shortages resulted in malnutrition and a lack of vitamin C, and antibiotics such as penicillin had not yet been discovered that could have controlled some secondary infections that proved to be lethal.

The 1918 influenza pandemic, caused by the H1N1 virus, provides valuable insights relevant to managing the COVID-19 pandemic. Markel et al. (20) demonstrated the profound impact of early intervention by comparing the disparate mortality rates in Philadelphia and St. Louis, each adopting different timelines for public health measures. St. Louis’s swift actions significantly reduced mortality, highlighting the importance of timely, decisive public health initiatives.

Moon (21) supported this observation, indicating that countries acting promptly before infection numbers surged achieved more favorable outcomes during the COVID-19 outbreak. The delayed pandemic declaration by the World Health Organization had adverse effects on early-affected countries, underscoring the critical role of timely action in public health crises.

The 1918 pandemic underscored the necessity of a robust public health infrastructure encompassing adequate staffing, medical equipment, and protective supplies. Nevertheless, many healthcare systems faced shortages of personal protective equipment and medical personnel during the COVID-19 pandemic, revealing gaps in pandemic preparedness.

Hobday and Cason (22) referenced the experience of Brooks Hospital near Boston to illustrate the potential benefits of open-air treatment, which resulted in lower mortality rates during the 1918 pandemic. While not universally applicable, this approach leverages natural light and improved ventilation, which could have been considered complementary to traditional healthcare settings during the COVID-19 pandemic.

The 1918 influenza pandemic imparts crucial lessons on early intervention, developing a resilient public health infrastructure, and exploring alternative treatment methods. These insights could have played a pivotal role in devising more effective strategies for managing the COVID-19 pandemic.

### Assessing the mistakes

A reflective examination of past deficiencies offers valuable insights into significant weaknesses within the healthcare system, particularly in preparedness, resource allocation, and communication. Strategic allocation of resources to address these identified areas holds the promise of bolstering resilience and fortifying capabilities for improved crisis management in subsequent instances. In the interest of conciseness, our attention will be directed toward the more contentious aspects of the COVID-19 pandemic.

### Reflecting on lockdowns

Lockdowns have emerged as a contentious yet often effective measure in controlling infectious diseases (23, 24). While some research underscores their efficacy, other studies advocate for a more nuanced strategy that considers factors such as timing and scale (25, 26). Notably, the broad application of lockdowns has come under scrutiny, particularly in the context of airborne diseases (27–29).

From an economic perspective, the consequences of stringent lockdowns have been substantial, resulting in business closures, job losses, and a global recession (30–32). In the United States, the estimated cost per prevented infection was $28,000, significantly impacting GDP (33).

Lockdowns also had notable repercussions on education and healthcare systems. These measures exacerbated educational inequalities, (34) and delays in non-COVID medical treatments were prevalent (35). The healthcare sector faced strains, particularly in terms of shortages in supplies and personnel, disproportionately affecting vulnerable communities (36). The interruption of educational activities impacted the quality and accessibility of learning, particularly in under-resourced regions (37).

Considering the social and economic trade-offs associated with lockdowns, a more nuanced, context-specific approach is imperative for future pandemic management. This approach should encompass financial assistance, mental health support, and targeted public health interventions. For instance, strategies effective in essential sectors, such as reducing public transport occupancy, could be adapted to other sectors to alleviate economic pressures. Telemedicine holds promise as an alternative for healthcare delivery. The psychological impact of lockdowns, particularly on vulnerable populations, should not be underestimated. Interventions such as open-air visits may help mitigate some of these emotional costs (38). The approach to social distancing and lockdown measures requires a balanced consideration of multiple factors, encompassing public health, economic, and psychological well-being.

Although some assert the effectiveness of cloth face masks in preventing the airborne transmission of COVID-19, (39, 40) multiple studies have found them to be ineffective in this regard (41–46) and have highlighted adverse effects associated with prolonged usage.

The regrettable outcome of this resulted in a deviation from established pandemic protocols, wherein fear was disseminated, fostering an unscientific perspective that characterized individuals as bio-hazards.

### Reflecting on cigarette and alcohol bans

The approach to social distancing and lockdown measures requires a balanced consideration of multiple factors, encompassing public health, economic, and psychological well-being (47). However, these bans encountered criticism due to their perceived lack of well-founded rationale, especially given that existing lockdown protocols already restricted social interactions.

Several factors, including concerns about compromised immune systems and the potential for viral transmission through shared cigarettes, influenced the decision to prohibit the sale of these products. In South Africa, implementing such a policy led to cultural reactions, including the creation of a widely circulated parody song (48).

From a financial perspective, these bans had notable repercussions. Substantial revenue losses were observed, including foregone sin taxes, value-added taxes, and related income. Furthermore, illicit markets emerged for these banned items, exacerbating public health risks due to the poor quality and potential toxicity of the products. The bans also unintentionally gave rise to health issues, such as withdrawal symptoms and associated mental distress, for which sufficient addiction management support was often lacking.

An alternative strategy could have focused on educational initiatives aimed at informing the public about the specific health risks associated with smoking and alcohol consumption during the pandemic. Given that existing lockdown measures and restrictions on social gatherings were already in place to address many of the issues targeted by the substance bans, a more nuanced approach, incorporating public education, might have offered a more effective and less detrimental solution.

### Reflecting on alternative treatment strategies and the misinterpretation of evidence-based medicine

In the first year of the COVID-19 pandemic, healthcare professionals explored various treatment options using repurposed drugs. By December 2020, a body of literature had emerged, comprising *in vitro* studies, case series, and observational research, which collectively suggested the potential efficacy of these drugs in treatment protocols. However, with the introduction of vaccines at the end of 2020, a media campaign arose that largely discredited the use of therapeutics. This campaign cited the absence of large randomized controlled trials (RCTs) and dismissed existing literature as lacking in quality.

Over decades, medical education has perpetuated the notion that research not conforming to the RCT framework is of inferior quality. The evidence-based medicine (EBM) pyramid concept has contributed to this perception, implying that research quality progresses linearly from observational studies at the base to meta-analyses at the apex. However, this presents a misleading viewpoint, as the pyramid primarily signifies increasing certainty levels, not necessarily research quality. Moreover, it neglects significant categories of research, including *in silico*, *in vitro*, and *in vivo* studies.

To rectify this misconception, Aldous et al. (49) introduced an alternative model known as the T-EBM Wheel. This model offers a more nuanced and adaptable framework for evaluating various forms of evidence, particularly valuable in the early phases of pandemics. Unlike the conventional EBM pyramid, which emphasizes a hierarchical structure, the T-EBM Wheel provides a more practical guide for clinicians and policymakers. It is important to note that the T-EBM Wheel builds upon the principles of the traditional hierarchical model and, as such, complements this established method.

This innovative model argues for a departure from traditional hierarchical models, advocating for an inclusive approach that accommodates multiple forms of evidence and embraces real-world clinical observations. In particular, it facilitates the exploration of repurposed drugs in pandemic contexts, potentially offering effective early treatment alternatives.

### Reflecting on the lack of diverse thinking

The response to the COVID-19 pandemic has laid bare deficiencies in leadership and policy-making, notably regarding the limited diversity within decision-making groups. Initially, the global response was predominantly orchestrated by clinicians and bolstered by government officials. This narrow focus overlooked valuable contributions from social scientists, economists, legal specialists, and pure scientists, resulting in a restricted range of solutions and insufficient consideration of the broader societal impacts. It is essential to acknowledge that the importance of early involvement of Public Health specialists during a pandemic is not in dispute.

The proliferation of misinformation in the media and information blackouts due to government censorship (50–56) stifled dissenting scientific voices. This clinician-centric approach failed to account for diverse community needs, economic factors, and broader human rights concerns. For example, the initial emphasis on medical interventions like testing and vaccines failed to consider the social and economic repercussions. Vulnerable, low-income communities with limited access to healthcare were disproportionately affected. As circumstances evolved and trust eroded, it became evident that leadership needed to adopt a more comprehensive perspective.

The ivermectin (IVM)-Wheel introduced by Aldous et al. (49) illustrates that by 2022, there were discernible positive indications of efficacy evident across all categories of reports. Statistically positive findings could have provided valuable insights for using IVM-based treatment protocols. The positive results and inconclusive findings with positive signals could have served as foundational information for developing new IVM-based treatment strategies. Conversely, negative results would have indicated areas of treatment regimens requiring modification or avoidance.

Even flawed studies could have offered insights into aspects of treatment protocols that warranted cautious consideration. In cases where positive findings contradicted negative conclusions, such discrepancies could have pointed to components of treatment protocols that yielded positive effects.

**Figure 1:**
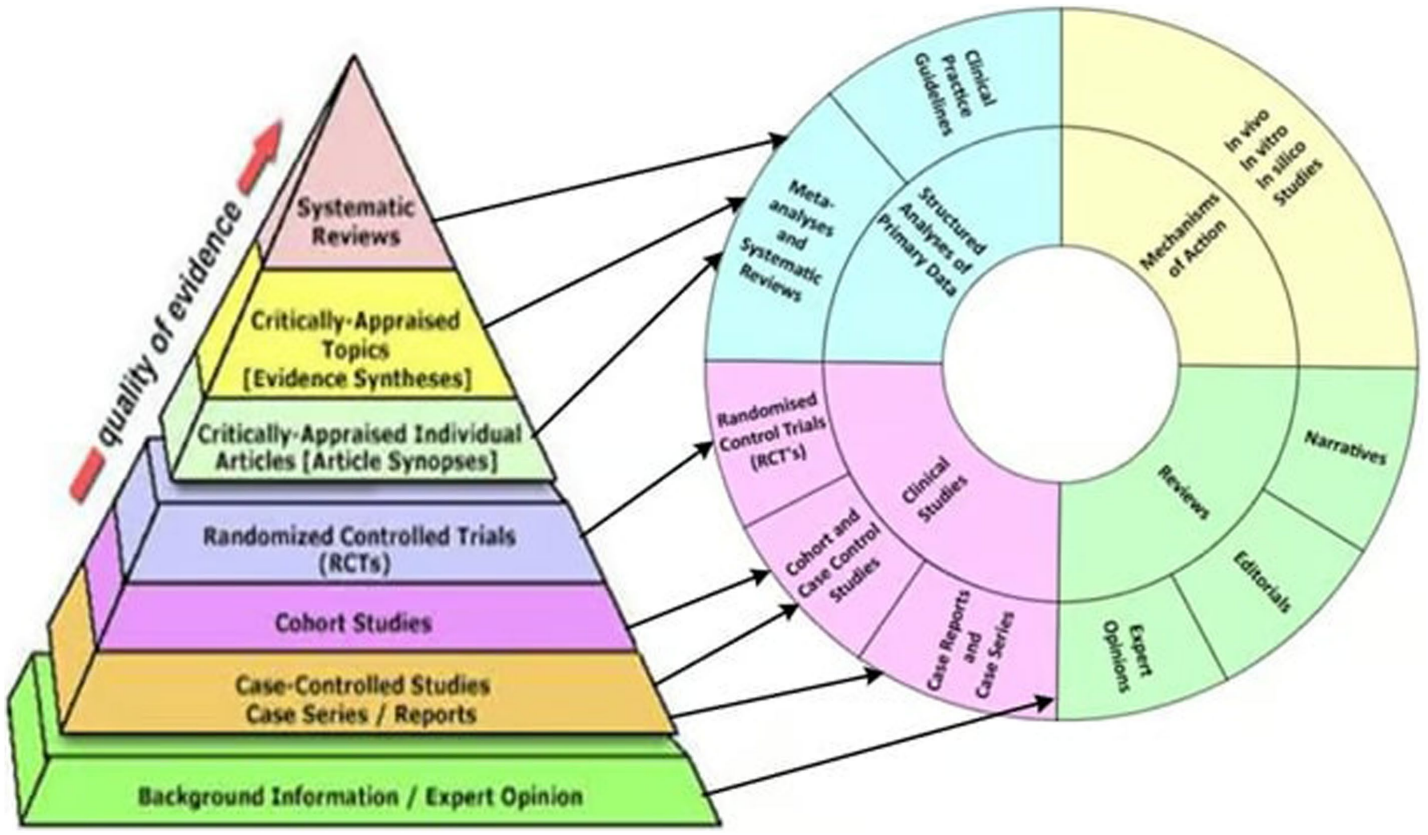
Correspondence of traditional evidence-based medicine pyramid with the two inner rings of the T-EBM wheel. Arrows show correspondences between levels of the pyramid and sections of the rings.

The use of an IVM T-EBM Wheel (e.g., Figure 1) by decision-makers in the early stages of the pandemic could have played a pivotal role in expediting the mitigation of COVID-19. This underscores the importance of incorporating such frameworks into future pandemic response strategies.

For a more effective and equitable pandemic response, diverse disciplines must be included:

1. Social Scientists: They bring insights into human behavior, social dynamics, and cultural influences, which can improve the effectiveness of public health measures like vaccine distribution and adherence to safety protocols.
2. Legal Experts: They can guide the legal frameworks essential for a pandemic response, ensuring that measures are constitutionally sound and respect individual liberties.
3. Economists: They can assess the economic impact of interventions and recommend strategies to mitigate economic damage while ensuring equitable response.
4. Pure Scientists contribute to understanding the virus at a fundamental level, providing data that can inform public health policies and potential treatment or vaccination strategies.
5. Multidisciplinary Approach: Including experts from finance, health, humanities, and legal fields would lead to a more holistic understanding and effective control measures.

In the face of future pandemics, global leadership should make a concerted effort to prioritize diversity of thought. This entails actively seeking diverse perspectives, which will, in turn, develop more comprehensive strategies. These strategies should encompass enhanced health communication tailored to meet the diverse needs of various communities and the implementation of nuanced workplace safety measures.

Embracing a multidisciplinary approach is pivotal in achieving a more effective and inclusive response. Such an approach would be instrumental in addressing the multifaceted challenges of pandemics, spanning medical, social, legal, and economic dimensions. By incorporating a wide range of expertise and viewpoints, global leadership can better navigate the complexities of future crises and devise solutions that cater to the diverse array of challenges that pandemics invariably bring forth.

## Holistic management

Alan Savory’s Holistic Management principles, applied globally in agriculture and conservation for over 40 years, have effectively balanced economic, social, and environmental elements (57–59). The emergence of the COVID-19 pandemic sparked discussions on adapting these principles for comprehensive pandemic management (60). Moreover, the literature indicates a broader trend shifting from reductionist to holistic models in various fields, including economics, health, and environmental management (61–64). General Stanley McChrystal emphasized the inadequacy of reductionist leadership in modern organizations (65).

This proposal put forth that future pandemics should be addressed holistically. In this approach, multidisciplinary advisory panels would seek optimal solutions that consider economic, social, and environmental factors related to the pathogen. Any proposed intervention would undergo analysis in terms of its advantages and disadvantages concerning these three critical dimensions. It is evident that the approach taken during the COVID-19 pandemic exhibited shortcomings in terms of economics, (66, 67) social aspects (30, 68) and the environmental context of the virus. For instance, isolating older adult individuals indoors instead of allowing them outdoor exposure had devastating effects (69).

Early evidence, including findings from the Diamond Princess outbreak, indicated that a significant majority of individuals (>85%) were asymptomatic to the virus (12, 70, 71). It is now known that asymptomatic individuals are less infectious than initially reported (72, 73). Despite such data, strict lockdowns were enforced globally, neglecting the potential for more nuanced strategies. As demonstrated during the 1918 Spanish flu pandemic, the impact of early intervention is crucial (20). In some instances, like South Africa, stringent lockdowns failed to contain the virus due to the widespread use of minibus taxis (74). By March 2022, over 98% of the South African population had been exposed to COVID-19 (75). In contrast, Sweden’s more balanced approach, (76, 77) resulted in better outcomes to those observed in the UK, which also opted for a hard lockdown (78).

Herein we argued that censoring dissenting views are counter productive. A prominent example of a dissenting voice from during COVID-19 is that of Dr. David Martin whose views were portrayed as outlandish. Although we are neutral about his claims, it remains to be seen to what extent the revelations by Dr. David Martin about the origin and handling of the COVID-19 pandemic hold true (79).

## Conclusion

Centralized decision-making during the pandemic, primarily led by select scientists (pandem-icons) who self-appointed as the sole voices of reason, resulted in a narrow, reductionist approach. This approach neglected critical factors, such as economic and social impacts, ultimately leading to suboptimal outcomes (80). The absence of multidisciplinary advisory panels perpetuated these limitations at considerable costs (30, 81).

Concerns have emerged regarding legal immunities granted to vaccine manufacturers by global governments and entities like the WHO, raising questions about the potential for expedited processes at the expense of safety (82, 83). Even though the WHO officially declared the end of the pandemic on May 5th, 2023, there is still a need for a broader discussion on the extent of governmental influence. To address these issues, it is recommended to establish multidisciplinary pandemic advisory panels rooted in Holistic Management principles. The adoption of the “Totality of Evidence-Based Medicine (T-EBM) Wheel” approach (49) aims to provide a nuanced understanding of various factors, promoting more inclusive strategies.

Looking ahead, fostering transdisciplinarity, robust debates, and transparent collaboration is crucial. Societies worldwide are encouraged to actively engage in discussions that delineate the boundaries of governmental power, fostering a collaborative effort to chart a pathway characterized by informed choices. This approach seeks to secure our civil liberties and collective well-being. Our collective responsibility should aim to develop evidence-based and holistic responses to pandemics, aligning with a commitment to global well-being and preparedness for future challenges.

## Data availability statement

The original contributions presented in the study are included in the article/supplementary material, further inquiries can be directed to the corresponding author.

## Author contributions

CA: Conceptualization, Writing – review & editing. HK: Conceptualization, Writing – review & editing.
